# Monitoring the Dutch Solid Start Program: Developing an Indicator Set for Municipalities to Monitor their First Thousand Days-Approach

**DOI:** 10.5334/ijic.6508

**Published:** 2022-10-25

**Authors:** Joyce M. Molenaar, Inge C. Boesveld, Jessica C. Kiefte-de Jong, Jeroen N. Struijs

**Affiliations:** 1 Centre for Nutrition, Prevention and Health Services, Department of Quality of Care and Health Economics, National Institute for Public Health and the Environment, Bilthoven, The Netherlands; 2 Health Campus The Hague/Department of Public Health and Primary Care, Leiden University Medical Center, The Hague, The Netherlands

**Keywords:** Delphi study, indicators, first thousand days, integration of care

## Abstract

**Introduction:**

The Dutch Solid Start program aims to improve the collaboration between the medical and social sector to offer every child the best start in life. Municipalities form local coalitions of partners within the medical and social sector to support parents and children during the first thousand days. The aim of this study was to develop an indicator set for coalitions to monitor their local Solid Start program.

**Methods:**

A modified Delphi study with three rounds was carried out among Dutch experts in Solid Start practice, policy and research (n = 39) to reach consensus.

**Results:**

The indicator set included 19 indicators covering the three phases of the Solid Start program: preconception, pregnancy and after birth (up to two years). Prioritized indicators included both social and medical topics, among which poverty, psychological/psychiatric problems, stress, smoking, cumulation of risk factors, preconception care, low literacy, premature birth, and intellectual disability. Additionally, a development agenda was established with topics and indicators that lacked data or clear operationalization (e.g. stress, unintended pregnancy, loneliness).

**Discussion and conclusion:**

The developed indicator set enhances the conversation between policymakers, managers, professionals and other stakeholders about the local situation and developments in order to prioritize interventions and policies. Next, the indicator set needs evaluation to assess its usefulness.

## Introduction

**Figure 1:**
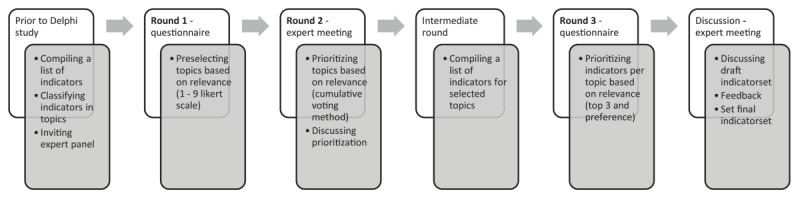
Schematic representation of the development and establishment of the indicator set.

**Figure 2:**
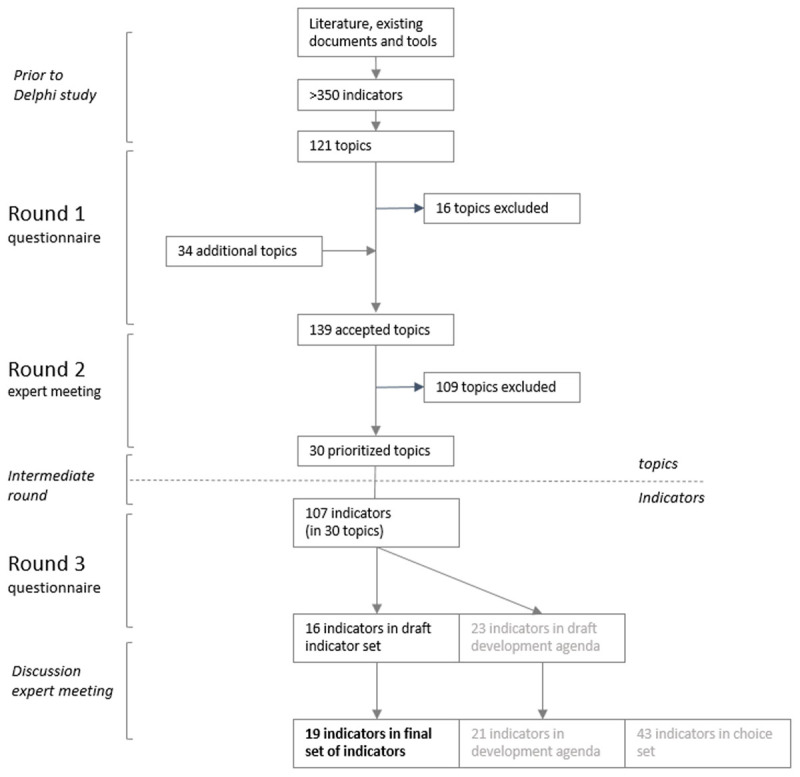
Flowchart of the selection of topics and indicators to monitor Solid Start on a local level.

Reducing perinatal health inequities and improving health outcomes for parents and children are high on the Dutch policy agenda since the early 2000s. Following alarming perinatal mortality and morbidity figures [1,2], several policy measures were taken to improve maternity care, including the establishment of maternity care networks [3], experiments with bundled payment for maternity care [4] and the development of the ‘Standard for Integrated Birth Care’ [5]. Over the years, the focus of the programs shifted from the medical sector more towards the social and public health care sector, as perinatal and maternal health is strongly influenced by the wider social, economic and cultural contexts of families [6,7]. For instance, a regional cross-sectoral approach to perinatal and maternal health, integrating the medical and social sector, was taken in the local ‘Ready for a baby’ program [8] and subsequent ‘Healthy Pregnancy 4-All’ programs [9,10,11]. These programs laid the foundation for the nationwide ‘Solid Start’ action program.

The Solid Start program was launched by the Dutch Ministry of Health, Welfare and Sport in September 2018 with the aim to give every child the best start in life by focusing on the first thousand days [12]. This period from preconception to the child’s second birthday is crucial for children’s further physical, mental and social development and is therefore regarded as a window of opportunity to improve population health [6,13,14]. The integrated approach of the Solid Start program combines medical and social services to offer better support during the first thousand days, specifically for parents in vulnerable situations. Consequently, the scope of integrated service delivery within the program is not limited to the health sector alone, but rather expanded to coordinate care and support also between the health and social sector (including public health) with its various organizations and providers (among which midwives, social workers, gynecologists, youth healthcare providers, debt counselors, and municipal officials). The Solid Start program is conceptualized and implemented over three phases: before pregnancy, during pregnancy and after birth (up to two years). Municipalities receive additional subsidies from the Ministry of Health to form local coalitions of partners within the medical and social sector, in order to tackle the region-specific challenges. Examples of region-specific challenges are unintended pregnancies, housing problems, domestic violence, and loneliness. This approach fits with the decentralization tendencies of social care in the Netherlands. Since 2015, the government has given municipalities new responsibilities in youth care, long-term care and income support, which cause local differences in policy implementation and outcomes [15]. Next to the subsidies, supportive methods were developed and offered to local coalitions. Examples include an analysis tool to map the current and desired situation and an overview of effective interventions (e.g. prenatal home visits and ‘Centering Pregnancy™’: group care during pregnancy). Moreover, local coalitions receive support to develop and implement their local coalition and related programs by Pharos, which is the Dutch Centre of Expertise on Health Disparities.

The Ministry of Health commissioned the National Institute for Public Health and the Environment (Dutch abbreviation: RIVM) to monitor the implementation of the Solid Start program. To this end, an indicator set including fifteen indicators was developed in a Delphi study with experts in 2019 [16] and reported annually in order to monitor the implementation of the nationwide program and to identify whether health outcomes improve. The indicator set reflects both processes (e.g. percentage of municipalities in which youth healthcare offers prenatal home visits) and outcomes (e.g. percentage of children born prematurely or with a low birth weight). In addition, the RIVM conducted a process evaluation to collect the experiences of those involved in the Solid Start program in order to provide further insight into factors that promote and hinder the implementation. The Ministry of Health uses the results of the monitor in combination with other data sources and expert opinions to determine whether goals are being achieved and to timely adjust policies. The results of the national Solid Start monitor showed that local coalitions evolve and formalize and that the majority of them also plan to monitor their local program, or have started to do so [17,18,19]. However, the local coalitions generally experienced a lack of insight into which indicators to include in their local setting, where to find the data for their municipality and how to make optimal use of it. Because the national indicator set was considered less suitable for monitoring on a local level, they expressed a need for a uniform indicator set to use within their local coalition. In 2021, the RIVM started a support program that is focused on monitoring Solid Start on a local level (for additional information about the support program and its relation with the Solid Start program and national monitor see Appendix A). Key elements of this support program include learning from and with other stakeholders (both within and between local coalitions) and sharing best practices within learning communities. The local coalitions that participated in the monitoring support program considered the development of a suitable indicator set the essential first step to stimulate monitoring on a local level.

In this paper, we describe our approach in developing an indicator set to monitor the Solid Start program in Dutch local coalitions and we present this indicator set. The indicator set can be used by local coalitions to enhance the conversation between policymakers, managers, professionals and other stakeholders about the local situation and developments in order to prioritize interventions and policies. This can help to strengthen and promote integrated service delivery.

## Methods

### Design and procedure

Within this mixed-methods study, we used a modified Delphi technique as a structured method to reach consensus on an indicator set to monitor Solid Start on a local level [20]. This commonly used approach in health research is suitable to synthesize knowledge from various experts with a different background or geographical location [21]. Our study had several iterative rounds of self-administered questionnaires and expert meetings (Figure 1). The study was conducted between March and June 2021.

### Prior to Delphi study

The study started with compiling a list of indicators originating from existing monitoring tools or documents from local coalitions, scientific and grey literature, and the indicator set used in the national Solid Start monitor [16,22,23,24,25,26]. The list of possible indicators was long (in a first endeavor >350) because the scope of the first thousand days is comprehensive. As this was expected to be a burden to the participants, we decided to first select topics instead of indicators directly. One researcher (JM, health scientist) categorized and named the topics in line with existing monitoring tools and documents, and another researcher (IB, former midwife and advisor integrated maternity care organizations) cross-checked this. We categorized and named the topics based on the shared characteristics and common themes in indicators (e.g. indicators relating to a low household income, debts, receiving social benefits and stress due to finances were categorized into the topic ‘poverty’). Differences were discussed by three researchers (JM, IB and JS (expertise health economy)) until consensus was reached. We excluded topics that 1) did not have at least one operationalized indicator, or 2) exceeded the time period of the Solid Start program (i.e. beyond the first thousand days of life). Topics were classified in the three phases of Solid Start (preconception, pregnancy and after birth) with the reason to eventually get a sufficient number of indicators per phase. Some topics were relevant in more than one phase.

### Expert panel

The expert panel consisted of a heterogeneous group of experts involved in Solid Start activities and experienced with monitoring, geographically distributed over the Netherlands (i.e. both rural and urban areas in the northern, eastern, western and southern parts of the country). We aimed for a balanced representation of experts in practice, policy and research (purposive sampling), including managers of local coalitions, policy makers, policy advisors, epidemiologists, researchers, educators, primary and secondary healthcare providers (e.g. midwife, nurse, gynecologist, pediatrician) and social workers. We invited members of the monitoring support program (Appendix A) and their network (‘snowballing method’), and we recruited participants through social media, Solid Start newsletters and webpages, and personal invitation. Those interested received more information about the aim, design and voluntary nature of the study. The views of participants all received equal weight during the study.

### Delphi round 1: questionnaire

In an online questionnaire, the Delphi panel was instructed to rate 121 topics based on relevance to monitor Solid Start on a local level on a nine-point Likert-scale (1 = not relevant at all, 9 = highly relevant). We gave an example of a possible indicator for each topic for comprehensibility. In addition, experts were invited to comment on the topics or to suggest additional topics for each of the three phases in the open spaces of the questionnaire.

All ratings were analyzed by calculating the median score and level of agreement between experts, following the RAND/UCLA Appropriateness Method user’s manual [27]. Based on the median scores, topics were classified as either inappropriate (median range 1 – 3), uncertain (median range 4 – 6) or appropriate (median range 7 – 9)(Appendix B). Level of agreement was assessed by the IPR-score (interpercentile range, difference between 30th and 70th percentile) and the IPRAS-score (interpercentile range adjusted for symmetry). If the IPRAS is larger than the IPR, there is agreement among experts and if the IPR is larger than the IPRAS, there is disagreement.

We planned to 1) accept topics with median score ≥ 7 with agreement, 2) reject topics with median score ≤ 3 with agreement, and 3) discuss all other topics (median score 4 – 6 or without agreement) in Delphi round 2. However, round 1 resulted in a large majority ‘accepted’ topics and well exceeded the number of intended indicators. We therefore decided to prioritize these ‘accepted’ topics in the second Delphi round and rejected all other topics.

The experts’ suggestions for new topics were read and discussed by the researchers (JM, IB, JS) until consensus was reached on additional topics. New topics were combined or reformulated if necessary and added to Delphi round 2.

### Delphi round 2: expert meeting

The second Delphi round consisted of expert meetings to prioritize the topics using the cumulative voting method. Meetings were held online due to Dutch COVID policy restrictions and we organized three separate smaller meetings to encourage active participation during the online meetings. The meetings of +– 120 minutes were recorded. Experts were first informed about the results of Delphi round 1. Next, they were encouraged to prioritize topics by dividing 100 points at their own discretion. After the individual prioritization, experts entered their scores into an interactive program to aggregate scores of all participants in the meeting. We encouraged experts to reflect on these aggregated scores. After the discussion, experts were invited to reconsider their earlier individual scores again. This sequence was repeated for the three phases (preconception, pregnancy and after birth).

Subsequently, we aggregated all final scores and classified the topics from high to low sum scores. Within every phase (preconception, pregnancy and after birth) we searched for a sudden decline in sum scores as a natural cut-off point for prioritized topics. This led to a draft list of prioritized topics.

In addition, we transcribed the expert meetings verbatim and analyzed the data using MaxQDA. One of the researchers (JM) coded the data for considerations in the prioritization and requirements for the indicator set. Coding was checked by a second researcher (IB).

The researchers (JM, IB, JS) consequently checked the draft list of prioritized topics against the experts’ requirements for the indicator set. We checked whether the requirements were fulfilled or whether we should add lower prioritized topics to fulfill the requirements. At the end of the second Delphi round, we had a final list of prioritized topics.

### Intermediate round

Based on the final list of prioritized topics, we made a list of possible indicators for each topic. Indicators were derived from our previous list of possible indicators (prior to Delphi study) as well as suggestions made by experts during Delphi round 1 and 2. Indicators were reformulated or merged in case they were not clearly defined or overlapped, based on consensus between two researchers and in line with the other indicators (JM, IB). In the rare case that there was no indicator available in the mentioned sources for one of the topics, the researchers (JM, IB) formulated potential indicators based on comparable indicators (e.g. indicators for the same topic in other phases). For each indicator, we described its numerator, denominator, data source, and data availability.

### Delphi round 3: online questionnaire

The third Delphi round consisted of an online questionnaire to select and prioritize indicators. The experts received a list of possible indicators (including numerator and denominator) for each topic and were encouraged to 1) select a maximum of three indicators they considered suitable to monitor Solid Start on a local level, and 2) indicate their number one preference. In case only one possible indicator was presented, experts were asked whether or not they considered that indicator suitable. The experts were also invited to add comments.

For each indicator, we calculated the percentage of experts that selected the indicator within their top three or as their preference. The scores and comments were discussed by the researchers (JM, IB, JS) in order to select at least one indicator per topic. In this process, the following conditions were considered: 1) Is there a clear preference towards one indicator? 2) Is data available for this indicator in nationwide data sources for every municipality? 3) Is the indicator sufficiently operationalized? If all conditions were met, the preferred indicator was added to the draft indicator set. We additionally prepared a ‘development agenda’ for topics and indicators that were clearly preferred, but lacked data in nationwide data sources or a clear operationalization. In this case, a lower ranked indicator for this topic with data-availability and sufficient operationalization was added to the draft indicator set.

### Discussion – expert meeting

In a final two-hour online expert meeting we presented the draft indicator set (including the ‘development agenda’) and asked experts for feedback. Specifically, we checked whether the set covers the various elements to appropriately monitor Solid Start on a local level. Experts were encouraged to share their thoughts in the meetings’ chatbox or by e-mail afterwards. Pressing issues were discussed directly. Based on the meeting minutes and written feedback, we finalized the indicator set.

### Ethical considerations

Following the Dutch Medical Research Involving Human Subjects Act (WMO), ethical approval was not necessary for this study (http://www.ccmo.nl), as we did not conduct medical-scientific research and participants were not exposed to treatment or required to follow a certain behavioral strategy. All participants gave written informed consent. In an information letter and at the start of each round or meeting, we stressed that participation was voluntary and confidential, and that data were processed anonymously.

## Results

### Participants

The expert panel consisted of 39 experts (Table 1). The full questionnaire to select topics (round 1) was completed by 39 experts and 28 experts joined the online expert meeting to prioritize topics (round 2). A total of 28 experts participated in the questionnaire to select indicators (round 3) and 21 experts were present during the final expert meeting. 18 experts joined during the full study.

**Table 1:** Characteristics of participants.


	TOTAL **	ROUND 1 – QUESTIONNAIRE	ROUND 2 – EXPERT MEETING	ROUND 3 – QUESTIONNAIRE	DISCUSSION – EXPERT MEETING

Total number of participants	**39**	39	28	28	21

Field of expertise					

Policy*	**22**	22	16	16	14

Practice*	**12**	12	7	9	7

- Social sector	**4**	4	2	3	1

- Medical sector	**3**	3	1	1	1

- Both	**5**	5	4	5	5

Research*	**9**	9	7	6	4

Other* (e.g. providing support for collaboration and the formation of Solid Start coalitions in general)	**3**	3	3	3	2


* More than one field of expertise is possible. ** The same pool of 39 experts was approached in each round (e.g. the discussion was attended by 21 of these 39 experts).

Figure 2 shows a flowchart of the selection of topics and indicators during the study.

### Round 1 – questionnaire

The experts received 121 possible topics to rate. Out of these, 105 topics were selected (median score ≥7) and 16 topics were excluded (median score <7)(Appendix B). These excluded topics mainly concerned complications or medical risks during pregnancy or after birth (e.g. gestational diabetes and caesarian-section). Based on the experts’ suggestions, 34 topics were added. Some topics were completely new, but most were already mentioned in another one of the three phases (preconception, pregnancy, after birth). In total, 139 topics were selected for round 2.

### Round 2 – expert meeting

Experts prioritized topics within each of the three phases (Appendix B). For the preconception phase, the topic ‘poverty’ received the highest sum score. The topic ‘cumulation of risk factors’ received the highest sum scores for the phases of pregnancy and after birth. A decline in sum scores was clear in the pregnancy-phase after 10 topics (from 112 points to 96 points), but less clear for the other phases. We selected the prioritized 10 topics within each phase (a total of 30 topics, Table 2). Most topics belonged to two or three phases.

**Table 2:** Overview of the prioritized topics (n = 30)*.


	PRECONCEPTION	PREGNANCY	AFTER BIRTH (UP TO TWO YEARS)

Topics in all three phases*	Poverty	Poverty	Poverty

	Early detection by healthcare provider	Early detection by healthcare provider	Early detection by healthcare provider

	Health: psychological/psychiatric problems	Health: psychological/psychiatric problems	Health: psychological/psychiatric problems parents

	Health: stress	Health: stress	Health: stress

Topics in two phases*	Domestic violence (including screening)	Domestic violence (including screening)	

	Substance use: smoking	Substance use: smoking	

		Social network	Social network

		Cumulation of risk factors	Cumulation of risk factors

Topics in one phase	Preconception care	Care: multidisciplinary collaboration	Health outcomes child: premature birth

	Interventions (process indicators)	Unintended and/or unwanted pregnancy	Relation parent – child

	Low literacy		Health: intellectual disability parent

	Client characteristics: socioeconomic status		Child abuse and neglect


* The topics that occur in multiple phases are presented on the same row.

Experts mentioned multiple requirements for the final indicator set (see Appendix C for a description of all requirements and corresponding quotes). The indicator set should include indicators regarding both processes and outcomes, and both parents and children. Experts moreover wanted to include indicators that have the potential to be influenced (to identify early effects of policy) as well as indicators that show prevalence rates (to be used in making policy). The total indicator set should be balanced in terms of risk- and protective factors and in general it should provide a full picture of all relevant aspects. The indicator set should provide a starting point of the conversation within a cross-sector collaboration. Lastly, it was considered important that data are available for the indicators. No additional topics were added to the final indicator set based on these requirements, since the prioritized topics largely seemed to match these requirements.

### Intermediate round

For the 30 prioritized topics, 107 unique indicators were found by the research team in the different sources. The number of potential indicators per topic varied from 1 to 7.

### Round 3 – questionnaire

Based on the experts’ selection and prioritization, the preferred indicator was clear for 20 topics (Appendix B). 11 of these indicators lacked data and were added to the development agenda. As the ‘second best’ option, 5 lower prioritized indicators for the corresponding topics were added to the draft indicator set. The draft indicator set consisted of 16 indicators, the draft development agenda of 23 indicators.

### Discussion – expert meeting

In general, experts appreciated the draft indicator set. They mentioned a number of extra non-prioritized indicators, which were added to an additional ‘choice set’ in case data was available (Appendix B). This set complements the basic indicator set and allows local coalitions to use additional indicators (e.g. regarding educational level, single parent family, long-term low income) if they want to.

In reflecting on the indicator set, experts mentioned some conceptual considerations (e.g. indicators are often formulated as risks, while the reverse can be a protective factor). They also mentioned methodological considerations (e.g. indicators regarding children’s health at age two are currently missing and should be added when more youth healthcare data is available). Experts gave their consent to the indicator set provided that the set will be piloted in practice. Based on the experts’ feedback, the indicator set and development agenda were finalized.

### Final indicator set

Finally, 19 indicators could be selected to monitor Solid Start on a local level (Table 3): 7 in the preconception phase, 5 during pregnancy and 7 after birth (up to two years). Some examples are debts, psychological or psychiatric problems, late antenatal care, smoking during pregnancy, vulnerability during pregnancy and after birth, not receiving postpartum care, and preterm birth and/or low birth weight for gestational age (SGA). Appendix B describes the selected indicators in more detail. Data is available in nationwide data sources for all these operationalized indicators and can be presented at local (municipality) level.

**Table 3:** Selected indicators to monitor Solid Start on a local level (n = 19).


PRECONCEPTION

Percentage of women and men in the reproductive age with debts

Percentage of women and men in the reproductive age with psychological or psychiatric problems

Percentage of women and men in the reproductive age with stress

Percentage of women in the reproductive age who smoke

Percentage of families reached with a preconception consultation (preconception care)

Percentage of low literacy among young people (<30 years) without partner and children

Percentage of women and men in the reproductive age living in a neighbourhood with a low liveability score

**PREGNANCY**

Percentage of pregnant women with debts *

Percentage of pregnant women who have their first antenatal care visit after the 10th week of pregnancy *

Percentage of pregnant women with psychological or psychiatric problems

Percentage of women who smoke at some point during pregnancy

Percentage of pregnant women in a potentially vulnerable situation (3 or more risk factors to vulnerability)

**AFTER BIRTH (UP TO TWO YEARS)**

Percentage of children born in a family with debts

Percentage of families not receiving postpartum care (at home) after birth *

Percentage of children aged 0 to 2 years of whom one or both parents have psychological or psychiatric problems

Percentage of children born in a family in a potentially vulnerable situation (3 or more risk factors to vulnerability) *

Percentage of children with a preterm birth or with a low birth weight for gestational age (SGA) *

Percentage of children born in a family of which one or both parents have a mild intellectual disability

Number of out-of-home placements for children before the age of 2 (per 1,000) *


* These indicators are also included in the indicator set to monitor the national Solid Start program.

The development agenda consists of 21 indicators (Appendix B). These (preferred) indicators lacked data or a clear operationalization. Some examples are smoking before pregnancy, stress due to finances, unwanted or unplanned pregnancy, stress during pregnancy, loneliness among parents, secure bonding, abuse or neglect of children and stress with parenting.

## Discussion

In this paper, we present an indicator set to monitor the Solid Start program in Dutch local coalitions, and we describe how we used a modified Delphi technique to reach consensus. The final indicator set consists of 19 indicators, covering the three phases of the Solid Start program: preconception (n = 7), pregnancy (n = 5) and after birth (up to two years)(n = 7). These indicators are available in nationwide data sources and can be presented on local (municipality) level. The indicator set meets the requirements as mentioned by the experts; it contains indicators that cover both processes and outcomes, both parents and children, and both risk- and protective factors. Additionally, the indicator set reflects both medical and social factors. A development agenda was established with topics and indicators that were prioritized, but lacked data in nationwide data sources or a clear operationalization.

The indicator set covers the following topics: poverty, psychological/psychiatric problems, stress, smoking, cumulation of risk factors, preconception care, low literacy, socioeconomic status, premature birth, intellectual disability, and child abuse and neglect. The first four topics are presented in the indicator set for all three phases (preconception, pregnancy and after birth). In general, the social determinants of health [7,28] are represented in the indicator set (e.g. debts, low literacy and living in a neighborhood with a low livability score). Specific clinical aspects that belong to one group of care providers (e.g. caesarean section, a child’s hearing) are less present. Nonetheless, the indicator set reflects both medical and social care, which aligns with the aims of the Solid Start program. In comparison to the indicators used in the current national Solid Start monitor (Appendix D), there is some overlap (e.g. debts during pregnancy, preterm birth and low birth weight for gestational age) but also differences. For instance, the national monitor also includes indicators such as the percentage of municipalities that implemented the program ‘Not Pregnant Now’. These differences are arguably caused by the different purposes of both indicator sets. The indicators in the national monitor can be used to monitor and evaluate the nationwide implementation of the program, and to monitor health outcomes of parents and children on a national level. As the implementation and health outcomes vary between municipalities, the indicator set of the local monitor aims to enhance the conversation between policymakers, managers, professionals and other stakeholders about the local situation and developments in order to prioritize interventions and policies at a local level.

A development agenda was made with indicators and topics that lacked data in nationwide data sources or a clear operationalization. Among others, the topics and indicators on the development agenda were related to stress, unwanted or unintended pregnancy, (quitting) smoking before pregnancy, loneliness, early detection, secure bonding, and child abuse or neglect. Multiple indicators related to stress were prioritized: stress due to finances, stress during pregnancy and stress with parenting. There is growing scientific evidence that stress during pregnancy or parenting has long- and short-term consequences for children’s health and development [14,29,30]. The multidimensional concept of stress [31] may require different indicators. It seems, therefore, valuable to explore which topics of the development agenda should be prioritized to be incorporated in routine registries for the purpose of local monitoring.

There are, to the best of our knowledge, no other studies that used a Delphi technique to identify indicators for local monitoring of the full first thousand days (approach). There are, however, several previous studies that sought to describe indicators for aspects of the first thousand days, including antenatal care [32], obstetrical care [33], children’s health [34], birth center care [35], and maternal and newborn health [36] or care [25] during pregnancy, childbirth and the postpartum period. Next to that, we found several programs in other countries that were focused to the first thousand days, but the aims, scope and key-design elements of the programs and their evaluation differ [37,38,39,40,41]. These programs were often not directly comparable to the Dutch Solid Start program and not (yet) focused on supporting monitoring on a local level. Consequently, a comparison between our indicator set and indicators in the aforementioned studies is hampered, with the exception of a study from Sweden [26]. In this Swedish study, the researchers developed indicators, sub-indices and a summary index in order to support municipalities with monitoring children’s health. In comparison to our study, they also mentioned both risk- and protective factors and also selected indicators related to poverty, smoking and low birth weight.

### Strengths and limitations

A strength of this study is that the indicator set is developed based on the expertise of a heterogenic and balanced group of experts in policy, practice and research related to the first thousand days, who have an interest in using the set in daily practice [20]. The focus of the indicator set to the first thousand days, involving both the social and medical sector, is necessary for programs aimed at reducing health inequities as health outcomes are directly and indirectly influenced by both social and medical factors [6,9,42]. The experts exchanged information and expressed their views during two expert meetings, as done in previous Delphi studies [20]. We organized a meeting to discuss and prioritize topics (Delphi round 2) and a final expert meeting. We considered this final moment of reflection on the (draft) indicator set very important to increase the support and future uptake of the indicator set in practice.

However, this study also has several limitations. First, we selected indicators based on consensus without considering the scientific evidence for these indicators. This does not necessarily mean that indicators that were not prioritized are not valid and vice versa. For most indicators to monitor maternal and neonatal health, their level of evidence is not well described [25]. In general, the rare availability of evidence is one of the reasons to (partly) select indicators based on experts’ opinions in a Delphi study [20]. Another limitation was that not all indicators in the final set were the preferred option by experts as a consequence of limitations in data availability. Hence we included some ‘second best’ indicators and added the preferred indicators to the development agenda. Other limitations relate to the inclusion of experts. This depended on the availability and willingness of experts to participate within the study’s time period, and on the decisions of the researchers in how and who to invite. Moreover, we invited experts from practice, policy and research in both the social and medical sector. Making a clear distinction between and within those categories is not always possible, as multiple experts work at the intersection of the various fields of expertise (practice, policy and research) or in multiple sectors (medical and social). For example, managers of local coalitions can be categorized as working in both practice and policy, as well as within the medical and social sector. The inability to distinguish between the field of expertise and sector is however in line with the aims of the program (i.e. integrating service delivery across the medical and social sector). Therefore, we do not expect that this may have influenced the results. This is also reflected in our results, as the experts from different fields of expertise and sectors did not prioritize different topics and indicators. Additionally, some experts dropped out during the study period, but the three groups of experts from practice, policy and research were all well represented during the various rounds. In addition, we missed the perspective of parents themselves. Finally, due to the COVID-19 pandemic, we were unable to organize physical meetings. Our decision to organize three smaller online meetings hindered the exchange of information and considerations between all experts. However, since the results of each of the meetings were highly comparable, we expect little influence on the results.

### Future research and practice

Recently, the first indicators were quantified and presented to all municipalities in the Netherlands at www.regiobeeld.nl/kansrijke-start. In the future, we will further refine the website with additional indicators and new functionalities (among which maps with geospatial variation). In quantifying the indicators, we use nationwide observational data sources with routinely registered data, which are linked on individual level. In the last decade, the opportunities of linking observational data sources has increased at an enormous pace, which enhances the usefulness and applicability of the developed indicator set [43].

The indicator set has yet to be used and evaluated in practice, as we can only determine the feasibility through empirical testing. A previous systematic review concluded that not many published indicators for maternal and neonatal health are empirically tested for validity and feasibility [25]. Starting in 2022, we will evaluate and refine the indicator set in close collaboration with the participants of the monitoring support program (Appendix A) in order to stimulate the uptake and adoption in daily practice. During this process, we expect to also discover which indicators are most often used and how, also for indicators that are similar across two or three phases (e.g. debts before pregnancy, during pregnancy and after birth). Using the indicator set should not be a one-time action, because the strength of using indicators for monitoring in municipalities is the comparison with previous comparable figures [26]. In the future, the indicator set will be refined because of new developments, changing demographics, new evidence and increased data-availability. In reflecting on the use of the indicator set, it is also important that we pay attention to questions about obtaining and presenting the data.

In the coming years, the topics on the development agenda will be prioritized and addressed in collaboration with national parties and local professionals. Central in this process is the formulation and operationalization of indicators and the expected increase of data-availability. Next to the indicator set and development agenda, the choice set with extra, non-prioritized indicators is also publicly shared (including where to find the data) for local coalitions to use.

### Relevancy

We consider our study scientifically relevant as it increases our understanding of relevant indicators for Solid Start and of using a systematic approach in developing indicators for monitoring a cross-sectoral program. In addition, it is relevant for society, as we can directly benefit from the study results by using the indicator set in practice. In the Netherlands, the indicator set can be used by local coalitions in collaboration with local stakeholders to describe their population, to identify gaps in current processes, to make or adapt policies, to prioritize interventions, to monitor developments and to stress the importance of investing in the first thousand days. In this monitoring process, combining quantitative data with qualitative data about experiences, facilitators and barriers (in a mixed-methods approach) can help to interpret the quantitative data, gain more insight into processes and explore opportunities for improvement [44]. Using the indicator set in combination with qualitative data in a continuous learning cycle with local stakeholders can support an integrated approach that is adapted to the local context in Dutch municipalities. On an international level, the topics and indicators can potentially be a starting point for monitoring similar cross-sectoral programs into the first thousand days in other western countries [37,38,39,40,41]. Additionally, countries that aim to develop a supported and comprehensive indicator set to monitor a cross-sectoral program can learn from our systematic methodology of collaborating with experts with varying backgrounds. Using a co-creative process can increase the support, relevancy and therewith impact of the research project [45,46].

## Conclusion

In this study we present an indicator set for monitoring the Dutch Solid Start program on a local level, which will be used and evaluated from 2022 onwards. The indicator set consists of 19 indicators that reflect both social and medical factors. The indicator set can be used by local coalitions to enhance the conversation between stakeholders about the local situation and developments in order to prioritize interventions and policies. Using the indicator set for monitoring is a continuous process that supports the optimalisation and promotion of integrated service delivery across the medical and social sector at a local level. Ultimately, the indicator set contributes to the reduction of health inequities within the preconception period, during pregnancy and after birth in order to give each child a solid start.

## Additional Files

The additional files for this article can be found as follows:

10.5334/ijic.6508.s1 Appendix A. RIVM monitoring support program – ‘Learning Local Monitor Solid Start’.

10.5334/ijic.6508.s2 Appendix B. Results of Delphi round 1, 2, and 3, final local indicator set, choice set and development agenda.

10.5334/ijic.6508.s3 Appendix C. Considerations in the prioritization and requirements for the final indicator set.

10.5334/ijic.6508.s4 Appendix D. National indicator set.
