# Supplementary material for: Monitoring the Dutch Solid Start Program: Developing an Indicator Set for Municipalities to Monitor their First Thousand Days-Approach
Source: Int J Integr Care. 2022 Oct 25;22(4):8. doi: 10.5334/ijic.6508 (PMC9615606; doi:10.5334/ijic.6508)
Supplement: Appendix A. — RIVM monitoring support program – ‘Learning Local Monitor Solid Start’. [file ijic-22-4-6508-s1.pdf]

Appendix A. RIVM support program – ‘Learning Local Monitor Solid Start’

In 2021, the National Institute for Public Health and the Environment (Dutch abbreviation: RIVM) started a support program focused on monitoring Solid Start on a local level. Key elements of the support program include learning from and with each stakeholder (both within and between local coalitions) and sharing best practices. The program stimulates local coalitions to use monitoring as a tool to further develop and improve their local approach.

There are eleven coalitions Solid Start that participate in regular learning sessions. These coalitions already started to monitor their local Solid Start program at an early stage; before or soon after the start of the national program. During these regular learning sessions (four in 2021), the specific needs for support are identified. These needs for support are discussed during several theme sessions (five in 2021) that are accessible to a wider audience. Everyone involved or interested in (monitoring) Solid Start can participate: professionals in the medical and social domain (e.g. midwives, social teams), researchers, managers, representatives of local organizations, etcetera.

The development of an indicator set to monitor Solid Start was considered by the eleven coalitions as the essential first step to stimulate monitoring on a local level. Other themes that were covered during the support program in 2021 were: 1) gaining insight into vulnerability, 2) monitoring the collaboration between medical and social domain, and 3) using monitoring and evaluation to learn, for example by involving experts by experience (parents or future parents) in local monitoring.
