# Supplementary material for: Monitoring the Dutch Solid Start Program: Developing an Indicator Set for Municipalities to Monitor their First Thousand Days-Approach
Source: Int J Integr Care. 2022 Oct 25;22(4):8. doi: 10.5334/ijic.6508 (PMC9615606; doi:10.5334/ijic.6508)
Supplement: Appendix C. — Considerations in the prioritization and requirements for the final indicator set. [file ijic-22-4-6508-s3.pdf]

Appendix C. Considerations in the prioritization and requirements for the final indicator set

Indicators regarding both parents and children	• Indicators concerning parents' health and well-being are important to develop policies that can improve the environment in which children grow up<br>• Children are key within the first 1000 days program and child outcomes can reveal whether policy eventually has the desired effect<br>*"I think you need a good mix in that and not only include the characteristics of the parents and the family where the child grows up."*
Indicators regarding both processes and outcomes	• Process indicators indicate how care and support is currently provided. This information can be jointly discussed to learn from<br>• Outcome indicators can help to identify the status quo and to check whether measures have effects. This information can be used to adapt policies and to account for expenditures to the city council. It stresses the importance to invest in children's health/ the first thousand days<br>*"Initially, the process is of course most interesting, because that is where most will happen. But in 5 years I find poverty considerably more interesting because then I expect that what I have done in the process will have an effect on poverty."*
Indicators that have the potential to be influenced (e.g. through policy)	• The indicators' potential to be influenced (through policy or other measures) is key to show short term successes<br>*"I also see it as a good outcome measure: if you give extra help and support, this is often noticeable in the percentage of mothers who will breastfeed."*
Indicators that show prevalence rates to use in making policy; both overarching (red flag) and specific	• Indicators that cannot easily be changed are also important to include in the indicator set if it concerns prevalence rates necessary to determine policy<br>• Indicators showing a 'red flag' are important for monitoring since they provide a general picture and necessity to take measures<br>• Specific prevalence rates on risk- or protective factors indicate which measures to take or which challenges to tackle<br>*"Indeed, you cannot really change education level, but [...] if you know that there are many low-educated people, you will take different measures than if you know that your population mainly consists of higher-educated people."*
Balance between risk and protective factors	• Protective factors to vulnerability are often overlooked while they are very important<br>*"It is of course very much about risk factors and I think there is an opportunity to look more at protective factors."*
Indicator set should provide a full picture of all relevant aspects	• The indicator set should provide a full picture or all relevant aspects<br>*"It is important in the prioritization to have a TOTAL VIEW across the board - so that the prioritized topics/indicators in the various phases say something about physical / mental / social / financial-work / environment-living / relationship- parenting / background / support interventions (and for example not a lot of indicators on physical and none or little on mental [health])."*
Indicator set should	• Indicators that require the exchange of information in the local setting are required;

provide a starting point of the conversation in a cross-domain collaboration	Collaborative partners can learn and work together based on this information<br>• Preferably, indicators should not belong to individual care providers only, but cross domains<br>*“In any case, these are things you especially want to learn together.”*
Indicators with data availability	• Data should be (easily) available on a local level<br>*“For multiple topics, it's about whether they are available locally.”*
